# Evaluating Social Interactions Using the Autism Screening Instrument for Education Planning-3rd Edition (ASIEP-3): Interaction Assessment in Children and Adults with Fragile X Syndrome

**DOI:** 10.3390/brainsci10040248

**Published:** 2020-04-22

**Authors:** Lisa Cordeiro, Marcia Braden, Elizabeth Coan, Nanastasia Welnick, Tanea Tanda, Nicole Tartaglia

**Affiliations:** 1 Department of Pediatrics, School of Medicine, University of Colorado, Aurora, CO 80045, USA; Elizabeth.Coan@childrenscolorado.org (E.C.); Tanea.Tanda@childrenscolorado.org (T.T.); 2 Licensed Psychologist, Private Practice, Colorado Springs, CO 80903, USA; drbraden@marciabraden.com; 3 Developmental Pediatrics, Children’s Hospital Colorado, Aurora, CO 80045, USA; Nanastasia.Welnick@childrenscolorado.org

**Keywords:** fragile X syndrome, autism, social interaction, ASIEP, outcome measure

## Abstract

An efficient and direct measure of social interactions and autism symptoms is needed for fragile X syndrome (FXS) research and clinical care. The Autism Screening Instrument for Educational Planning-Third Edition (ASIEP-3) Interaction assessment is a brief standardized measure that quantifies social responses under different conditions. The feasibility and validity of the ASIEP-3 was evaluated in 26 males and 13 females with FXS, along with cognitive testing and behavior questionnaires. The videos were scored at 10-second intervals, and the observed behaviors were scored as an interaction, independent play, no response, or aggression. In total, 39/41 participants successfully completed the ASIEP-3 (age M = 14.4 ± 10.2), with a range of cognitive abilities (abbreviated IQ (ABIQ) M = 58.9 ± 17.3, median = 50), behaviors (Aberrant Behavior Checklist (ABC) Total M = 37.00 ± 27.3), and autism diagnoses (N = 22/39). Reliable administration was demonstrated by all team members. The mean coded behaviors included interaction (40.6%), independent play (36.8%), no response (21.1%), and aggressive behavior (<10%). The interaction score was negatively correlated with the Social Communication Questionnaire (SCQ) score (*p* = 0.037), and the profiles differed by autism spectrum disorder (ASD) diagnosis. The intraclass correlation coefficients (ICCs) ranged from 0.79 to 0.93 for master’s level and above. Administration of the ASIEP-3 was feasible for FXS across sex, age, ability, and behavior ratings by a trained research team. Reliable scoring required advanced training in the assessment of social development and FXS experience. The scores correlated to ratings and diagnoses of ASD. The ASIEP-3 shows promise to reliably index social interactions in FXS.

## 1. Introduction

Fragile X syndrome (FXS) is the most common inherited form of intellectual disability (ID) and the most commonly known genetic cause of autism. It is caused by a trinucleotide expansion (CGG) of greater than 200 CGG repeats in the 5′ untranslated region of the fragile X mental retardation 1 gene (*FMR1*) located on the X chromosome and occurs in approximately 1 per 4000 males and 1 in 8000 females [1]. Individuals are normally categorized based on the size of the CGG repeat expansion, in which normal alleles have 5–44 CGG repeats, premutation alleles have 55–199 CGG repeats, and full mutation alleles have >200 CGG repeats. This expansion leads to a reduction or elimination of the *FMR1* protein (FMRP) production, which is essential for normal brain growth and function [2]. Variability in the functioning of the *FMR1* gene (X-activation ratio in females, methylation differences, FMRP expression, etc.) leads to a heterogeneous phenotype among both males and females with FXS [3].

Understanding the phenotypes of individuals with FXS is crucial when choosing assessments that will yield meaningful results in clinical trials. The developmental and behavioral profile of males and females with FXS is well documented. The spectrum of phenotypic expression of FXS in males includes early developmental delays and intellectual disabilities ranging from mild to severe (with approximately 10% exhibiting a borderline IQ) due to decreased methylation of the CGG expansion or mosaicism [4,5]. As an X-linked condition, females are typically less cognitively impaired than males, but approximately 30% have mild ID, and another 50% have learning impairments most commonly related to visual–spatial skill deficits and math learning disabilities [6,7]. The rates of speech-language disorders, repetitive speech, and both gross and fine motor deficits are also increased. The behavioral phenotype of FXS in both males and females is variable but also commonly includes anxiety, ritualistic behaviors, and behavioral outbursts that can include aggressive behaviors and/or self-injury, as well as attention deficit hyperactivity disorder (ADHD) symptoms, including a short attention span, hyperactivity, and impulsivity [8,9,10,11,12]. Social difficulties are common as a result of these cognitive and speech deficits and anxiety, but other symptoms, including gaze aversion, social communication deficits, abnormal sensory reactivity, motor stereotypies, and ritualistic behaviors, may lead to a co-occurring diagnosis of autism spectrum disorder (ASD). Females with FXS can show higher rates of anxiety, social withdrawal, avoidance, and an increased risk for depression compared to typical females [13,14,15].

### 1.1. Autism Spectrum Disorder in FXS

FXS accounts for an estimated 1% to 6% of all ASD cases. However, many individuals with FXS are co-diagnosed with ASD. Autism and FXS are typically diagnosed at separate times, and many are diagnosed with autism, which leads to genetic work-up and a subsequent diagnosis of FXS. Alternatively, some with a diagnosis of FXS identified due to early developmental delays or a known FXS family history are aware of their high risk and seek out ASD testing and a diagnosis after their FXS diagnosis. Depending on the research criteria and assessment measures used, studies have reported that 30% to 54% of males with FXS meet the diagnostic criteria for ASD compared to 16% to 20% of females [16,17,18,19,20,21,22,23]. Studies evaluating the features differentiating ASD in FXS have described lower verbal-IQ, receptive language skills, and theory of mind performance [24], as well as more significant behavioral problems [25]. Other phenotypic features of FXS, such as adaptive behavior, cognition, and repetitive behaviors, were not found to differentiate between young children with and without autism [26].

Though there is symptom overlap, professionals believe that the presentation of autism in FXS often calls for a different approach for evaluation and intervention compared to idiopathic ASD, primarily due to strengths in social interest and motivation, as well as increased symptoms of hyperarousal and anxiety. Studies delineating the unique features of ASD in FXS compared to idiopathic ASD have shown more prominent social withdrawal, higher levels of anxiety, and less intense repetitive and restricted behaviors [21,27,28].

### 1.2. FXS Clinical Trials and Outcome Measures

FXS is also one of the first single-gene disorders causing neurodevelopmental disabilities for which extensive study of the neurobiological mechanisms of the disease in cellular and animal models has been possible. The enormous progress in basic and translational FXS research has identified many neuronal targets and has facilitated early clinical trials of new treatments specific to the underlying disease [29,30]. A number of these agents (e.g., Arbaclofen and AFQ056) have shown benefits in open-label and early phase trials [31,32,33], but it has been challenging to meet primary behavioral outcomes in larger phase 2b and 3 trials. Challenges to meeting these outcomes within the context of clinical trials include multiple complex issues such as dosing, age of subjects, length of treatment, placebo effects, and primary outcomes of the target for the drug’s effect [34,35,36]. Largely, these trials have been limited to behavioral outcomes due to the necessary short length of placebo-controlled trials, as cognitive measures are not designed and less likely to show changes over short time periods. Other problems have included a poor availability of outcome measures and biomarkers across the FXS phenotype and a lack of well-validated cognitive measures, especially in the large subset of individuals with FXS with very limited language skills and low cognitive abilities. These challenges have contributed to an over-reliance on subjective outcome measures that are based solely on parental reports [37,38,39].

A need for new and validated outcome measures for FXS research trials was identified following challenges with the initial treatment trials [37]. The CDC FORWARD Component-C projects (RFA# U01DD001190) were funded with the goal of evaluating new outcome measures in FXS and documenting the longitudinal trajectory of FXS cognitive and behavioral profiles over one’s lifespan. Progress has been made over the past 5 years in the Fragile X field by evaluating more objective outcome measures that do not involve parental reporting, including measures of attention [40], expressive language [41,42], and executive functioning [43,44]. Eye tracking technology has been used to examine aspects of social attention while viewing videos [45,46], as well as in a naturalistic setting [47]. While these and other new measures have been studied, the identification of measures that directly and efficiently assess and quantify the different aspects of social interaction skills remains lacking [38].

### 1.3. Rationale for the Study

Differences in social development and the features of ASD are a core part of the FXS phenotype and an important domain for measurement in FXS clinical trials. The current measures of social development and ASD symptoms most commonly used in FXS research are parent-report questionnaires, such as the Social Communication Questionnaire (SCQ) or the Social Responsiveness Scale—2nd Edition (SRS-2). The SCQ was designed as a screening tool for identifying children who need further evaluation for ASD, and the SRS-2 was designed to capture different domains of social skills and the broader autism spectrum. While the SCQ and SRS-2 have been re-evaluated with new scoring algorithms and cut-offs in FXS [48], both continue to have limitations as parent-report measures, with subjective scores that are not derived from direct interaction with the participant.

Many FXS trials have included a direct assessment of ASD symptoms using the Autism Diagnostic Observation Schedule, 2nd Edition (ADOS-2), as a method of documenting the presence or absence of autism. The ADOS-2 is a semi-structured, standardized assessment of communication, social interaction, play, and restricted and repetitive behaviors and is considered the gold standard instrument in the diagnosis of ASD [49]. The examiner selects one of five different ADOS-2 modules depending on the age and expressive language level of the child, and each module is scored using different algorithms to identify scores in the No ASD, Autism Spectrum, or Autistic Disorder categories. Further algorithms are used to assign an ADOS Comparison score, which is used to relate the level of impairment compared to others of their age and language level. While the ADOS-2 is commonly used as a component of ASD clinical evaluation in individuals with FXS and is helpful for classification into diagnostic categories (ASD vs. no ASD), it was not designed to measure change over time. Specifically, the ADOS-2 uses very specific scoring criteria to assess the quality of various communication and social interaction skills and the presence of restricted or repetitive behaviors. This limits its ability to be used as a longitudinal quantitative measure of social development, as the selection of the appropriate module to be administered is determined by expressive language level and chronological age and scored using different algorithms. In addition, the scoring of the ADOS-2 involves rating the individual’s behaviors according to the quality of the skill shown for the majority of items and does not generally document instances or the frequency of social interactions. Therefore, comparing scores over time may be incapable of capturing the same components of symptomatology, as the social expectations and scored items differ from module to module, and increased instances of social interaction are not comprehensively captured using this tool. In addition, obtaining research reliability for ADOS-2 administration and scoring is very time-intensive and thus expensive for research trials. The length of the ADOS-2 (45–60 min) is also a burden to participants and researchers during time-sensitive and behaviorally taxing appointments. Thus, identification of a more efficient measure that directly evaluates social interaction, is able to monitor change over time, and can be used across age ranges and phenotypic involvements is a clear area of need within FXS research.

In this study, we sought to evaluate a new potential measure for use in FXS clinical trials and clinical research that would provide a direct and efficient measure of social functioning designed to evaluate change over time. A measure called the Autism Screening Instrument for Educational Planning-Third Edition (ASIEP-3) was utilized in the original FXS research documenting autistic behaviors in individuals with FXS [50,51] and continues to be used clinically for FXS and autism in educational and clinical settings. In this project, the ASIEP-3 Interaction Assessment component was examined as a potential direct measure of social interaction that is feasible from a very young age through adulthood [52]. Beyond its use as an outcome measure for trials, we also considered the characteristics of the measure and our results as a potential tool in therapy or in educational settings.

The ASIEP-3 is a five-part standardized measure designed to aid in the differential diagnosis, educational treatment planning, and monitoring of symptoms and progress over time. It includes (1) the Autism Behavior Checklist, (2) the Educational Assessment, (3) the Vocal Behavior Assessment, (4) the Prognosis of Learning Rate and (5) the Interaction Assessment. Each of these five components is independent of the other with distinct record forms and scoring. The Autism Behavior Checklist subtest is a checklist of non-adaptive behaviors. The Educational Assessment is a routine probe of a child’s functioning level in five areas: staying seated, receptive language, expressive language, body concept, and speech imitation. The Vocal Behavior Assessment includes four characteristics used to analyze the spontaneous speech of autistic children: repetitiveness (stereotypy), non-communication (social relating), intelligibility (expressive delay and deviance), and babbling (non-meaningful vocalizations). The Prognosis of Learning Rate is an assessment used to obtain an indication of a child’s ability to learn a new task in a direct teaching format given reinforcement. The Interaction Assessment is designed to look at relating passive/no initiation and direct cues in a play setting through direct observation of the child’s responses to an adult, thereby providing active modeling.

**Figure 1:**
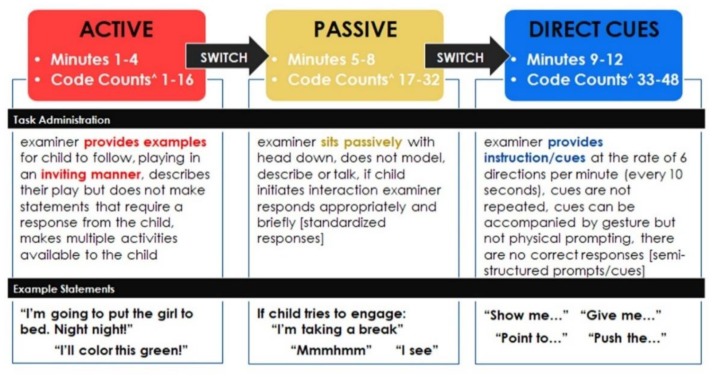
ASIEP-3 Administration Diagram. Active, Passive, and Direct Cue Phases: Task Administration Description and Examples of Examiner Statements. ASIEP-3: Autism Screening Instrument for Educational Planning- Third Edition.

The Interaction subtest evaluates and quantifies social interaction skills, including spontaneous social responses, acknowledgement of direct requests, and the individual’s ability to interact under different conditions during a 12-min video recorded session with an examiner. See Figure 1 for more details and an additional description in the Methods section below.

The ASIEP-3 was designed to measure change over time, making it ideal for evaluating change in a clinical trial setting or for longitudinal data collection. While experience with assessment and working with children with disabilities is important when training to administer this measure, this measure is much simpler to administer than the ADOS-2, much shorter, and does not have complex requirements for research reliability in administration or scoring. The ASIEP-3 is standardized for ages two through thirteen, which allows for the observation and assessment of a very young age group that is often missed by the current outcome measures used in this population. While standard scores are not available beyond thirteen years or below two years old, the raw scores and percentages can be evaluated across one’s lifespan using materials adapted for an older age group (i.e., magazines and iPods instead of toys). This adaptation has been useful for FXS in clinical use (M. Braden, personal experience) and has been endorsed by the authors of the ASIEP-3 who observed that the Interaction Assessment has been commonly used with populations over the age of thirteen whose developmental age is appropriate for this assessment (J.R. Arick, personal communication, March 31, 2020). The ASIEP-3 has been reported to discriminate between those with and without autism among those with an intellectual disability (ID) [53]. However, further evaluation of the ASIEP-3 as an outcome measure in FXS has not yet been attempted.

### 1.4. Aims of the Study

The aims of this study are to pilot and test the feasibility and clinical validity of the ASIEP-3 Interaction Assessment as an outcome measure designed to evaluate social interactions in FXS.

### 1.5. Research Questions

1. Can the ASIEP-3 Interaction Assessment be reliably administered and scored in a sample of individuals with fragile X syndrome across a broad range of ages and phenotypes?
2. How does performance on the ASIEP-3 Interaction Assessment vary in relation to age, gender, IQ, and behavioral functioning for individuals with FXS?
3. How do ASIEP-3 Interaction Assessment results compare between individuals with FXS and a clinical diagnosis of ASD (FX+ASD) and those without ASD (FX−ASD)?

## 2. Methods

### 2.1. Study Design

#### 2.1.1. Recruitment

Participants were invited to participate in the Colorado Multiple Institution Review Board (COMIRB)-approved Component C study (COMIRB# 15-1538) evaluating outcome measures and the longitudinal phenotype of FXS across one’s lifespan. This study was carried out at the University of Colorado and Children’s Hospital of Colorado (CHCO) Denver Fragile X Clinic. Inclusion criteria for the study included being a male or female with FXS, being thirty-one days to sixty-five years old, and already being enrolled or having agreed to enroll in the Fragile X Online Registry with Accessible Research Database (FORWARD) project. All potential participants meeting the inclusion criteria were approached with the opportunity to participate in the study, as well as in the FORWARD study if not already enrolled. FORWARD began in 2012 and consists of a patient and family registry plus a longitudinal database that includes clinician- and parent-reported data about individuals living with FXS [54]. Both FORWARD and Component-C (CDC U01DD001190) are funded by the Centers for Disease Control and Prevention (CDC). Participants were recruited from the Denver Fragile X Clinic and via multiple national advocacy and support organizations for FXS. All participants or their parents signed a COMIRB-approved consent form prior to participation. Medical records and genetic testing results were reviewed to confirm a diagnosis of FXS.

#### 2.1.2. Measures

A comprehensive assessment battery was administered (dependent on age) to characterize cognitive skills, adaptive functioning, behavior, autism symptoms, and social interaction, as described below.

##### Stanford Binet Intelligence Scales, Fifth Edition (SB-V)

The SB-V is a standardized measure of cognitive functioning. The SB-V provides scores for verbal and nonverbal ability across five domains—Fluid Reasoning, Knowledge, Quantitative Reasoning, Visual-Spatial Reasoning, and Working Memory—through which a full-scale intelligence quotient (IQ) composite score is derived [55]. The SB-V has high reliability, internal consistency, and well-documented criterion and concurrent validity. The SB-V Abbreviated Battery IQ (ABIQ) is based on two routing subtests—one nonverbal (Object Series/Matrices) and one verbal (Vocabulary). In this study, deviation scores were derived using an age-dependent (within each population age band) z-score transformation previously described [56] to generate non-verbal IQ (NVIQz), verbal IQ (VIQz), and full scale IQ (FSIQz) deviation scores for those participants who were administered a complete SB-V test. As six males completed only the routing subtests of the SB-V, the abbreviated ABIQ is also reported.

##### Vineland Adaptive Behavior Scales 3rd Edition, Comprehensive Interview (Vineland-3)

The Vineland-3 is a comprehensive adaptive behavior measure that yields composite standard scores in the domains of Communication (receptive, expressive, and written adaptive language functions), Daily Living Skills (personal, domestic, and community skills), Socialization (interpersonal relationships, play and leisure time, and coping abilities), and Motor Skills (gross and fine motor skills) [57]. The Vineland-3 also yields an overall adaptive functioning score, referred to as the Adaptive Behavior Composite (ABC). The Motor Skills domain is optional, does not contribute to the ABC, and is normalized up to 9 years of age [57,58]. The Vineland-3 has excellent internal consistency in the Comprehensive Form, with coefficients ranging from 0.94 to 0.99 and test–retest reliability ranging from 0.64 to 0.94 [59]. The Vineland-3 Interview Forms were administered by in-person or phone interviews with parents or caregivers during clinical and research visits.

##### Aberrant Behavior Checklist-Community Edition (ABC-C)

The Aberrant Behavior Checklist is a caregiver rating scale developed initially to assess problematic behavior in individuals with intellectual disabilities [60]. The ABC-C includes five original subscales: Irritability, Lethargy, Stereotypy, Hyperactivity, and Inappropriate Speech. The ABC-C has excellent reliability and validity [60,61,62,63]. Previous studies on FXS suggest that the ABC-C has good test-retest reliability [64].

Modified subscales of the ABC-C for FXS that include six subscales have been published. These subscales are Irritability, Socially Unresponsive/Lethargy, Hyperactivity, Inappropriate Speech, and Social Avoidance based on factor analysis [65]. In the modified ABC-C for FXS, several items of the Hyperactivity subscale were shifted to the Irritability subscale. The sixth subscale, Social Avoidance, includes four items taken from the original Lethargy/Withdrawal subscale. Strong internal consistency for the modified factor structure, including the six subscales, has been demonstrated [66]. Several FXS studies have shown that the ABC-C distinguished males with FXS from males with FXS and co-occurring ASD [19,21,67].

##### Social Communication Questionnaire (SCQ)

The SCQ is a parent-report screener questionnaire comprised of forty yes or no forced choice questions that evaluate communication skills and social functioning in children ages four or older who may have autism or autism spectrum disorders [68]. The SCQ is an efficient way to obtain diagnostic information and screen for autism symptomatology [69]. The SCQ items align with content from the Autism Diagnostic Interview—Revised (ADI-R). A total score is interpreted by considering the cutoff points to identify those who may have autism and should be referred for a comprehensive autism evaluation.

##### Social Responsiveness Scale—Second Edition (SRS-2)

The SRS-2 contains sixty-five items that measure the severity of autism spectrum symptoms across multiple domains (i.e., social awareness, social cognition, social communication, social motivation, and restricted interests and repetitive behavior) as they have occurred in natural social settings over the past six months [70]. Raw scores are converted into T-scores (M = 50, SD = 10) for each subscale, as well as the SRS-2 Total score.

##### Autism Screening Instrument for Educational Planning—Third Edition (ASIEP-3)

Two evaluators are required to administer the ASIEP-3 Interaction Assessment. The first adult interacts with the child across three different interaction conditions, which are each four minutes in length, while the second adult video records the interaction and provides cues on when to switch phases. The interaction includes three different conditions of four minutes each that are always administered in the same order: the active modeling phase (basic parallel play modelled by the examiner), the passive phase (the withdrawal of engagement and attention by the examiner), and the direct cues phase (the examiner gives specific directives during standardized time intervals). Prior to administration, information related to the participant’s cognitive level and expressive and receptive communication skills is reviewed, so the examiner is prepared to use developmentally appropriate language. Inappropriate play materials or verbal interactions that are too complex for the participant may confound the assessment results. The interactions between the examiner and the participant involve developmentally appropriate toys and activities selected from a standard menu developed for the study through the use of the Interest Inventory described below.

The ASIEP-3 is standardized for individuals up to thirteen years old. We adapted this measure for older adolescents and adults, for whom toys can be substituted for other items such as magazines, puzzles, adult coloring books, and card games appropriate for the developmental age of the participant. Many of the materials used in the senior psychologist’s (MB) clinical practice were replicated for use with the older subjects in the study. The total administration time of the assessment is twelve minutes (three phases, each four minutes long) plus an additional five to ten minutes for rapport building and explanation to the participant.

**Figure 2:**
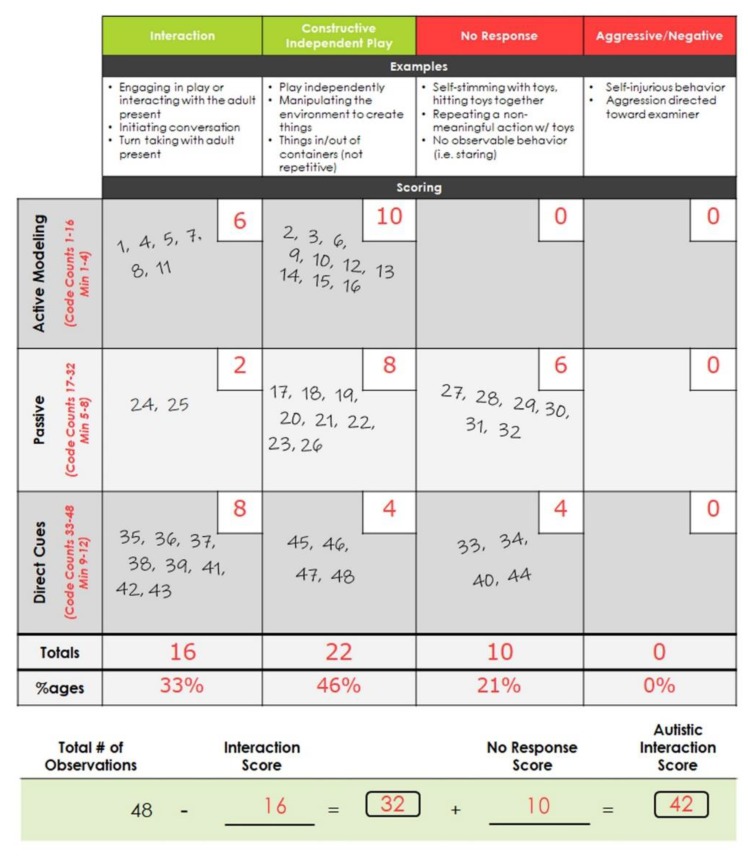
Example Autism Screening Instrument for Education Planning (ASIEP-3) Interaction Assessment Scoring Worksheet. While watching the assessment video, the behaviors are coded at 48 timepoints into one of four codes (Interaction, Constructive Independent Play, No Response, or Aggressive/Negative). ASIEP-3: Autism Screening Instrument for Educational Planning—Third Edition; Min: minutes; %ages: percentages; #: number.

Scoring occurs following the completion of the interaction and can also be completed by someone who was not present during the administration. The rater watches the session video while playing an audio overlay that marks ten-second coding intervals every fifteen seconds, with a total of forty-eight observations coded (16 observations in each of the three conditions). The rater rates the participant’s response for each interval into one of four different categories/codes: Interaction (responds, initiates, touches or complies), Constructive Independent Play (CIP) (independent play without social interaction), No Response (self-stimulation, self-abuse or no observable behavior or response), or Aggressive/Negative (tantrums, hits, cries, bites, etc.). Scoring yields counts and percentages for each of the four code types, as well as an overall Autistic Interaction Score (AIS). The AIS is derived by subtracting the total number of Interaction codes from forty-eight (the total possible number of all codes), and this value is then added to the total number of No Response codes. Therefore, a higher AIS indicates less interaction and more instances of no response. See Figure 1 for a further description of the phases and Figure 2 for an example of scoring, including the AIS.

##### Interest Inventory

All caregivers complete a brief Interest Inventory at each visit about the participant developed for this study (by MB), which is reviewed by the ASIEP-3 examiner prior to administration (see Figure S1). The Interest Inventory includes questions such as favorite activities, characters, foods and people, as well as any activities or topics to avoid. The Interest Inventory is reviewed to allow the staging of test materials and the planning of activities appropriate to the participant’s interests and developmental level. Due to the tendency for individuals with FXS to become intensely fixated on certain areas of interest, effort is made to ensure that the activities are of interest to the participant but that they do not cause them to become overly focused on one particular object or activity.

##### ASIEP-3 Administration Checklist

An additional checklist was developed for the study (by LC and NW) and completed by two to three research team members for each ASIEP-3 administration (See Figure S2). It was first completed by either the examiner or the recorder after ASIEP administration. It was completed a second (and sometimes third) time by each rater during their scoring process. This form included a checklist of play activities so that the materials provided and the materials engaged with could be tracked for each participant’s administration as a reference for subsequent administrations. Further, the ASIEP-3 Administration Checklist includes space for examiners to document specific details of the assessment and interactions related to imitation, triggers of aggression, details of any negative behaviors, and reactions to and behavior during the switch to the passive phase. The exploratory analyses of the ASIEP-3 Administration Checklist data are described below.

### 2.2. ASIEP-3 Administration Training

The research team included the senior clinical psychologist (MB), a clinical psychologist (EC), a psychometrist (LC), and 2 bachelor’s level research assistants (TT and NW). All evaluators (LC, TT, and NW) administered three practice ASIEP-3 Interaction assessments with one practice examinee from each of the three language groups (pre-verbal, non-verbal, and verbal) and were provided feedback. Further, the first twenty ASIEP-3 administration video recordings were systematically reviewed by the senior psychologist (MB) for all evaluators. Training sessions with members of the research team were held to separately teach the administration and scoring protocols. These training sessions involved quizzes to assess the learned information, as well as a review of the video examples and live-action roleplay using prepared participant scenarios developed for this study (LC). The examiners administering the ASIEP-3 were specifically trained to match the language level of the child during administration. For nonverbal individuals, the examiner was instructed to use one- to two-word phrases. It was also important to train the examiner in child-centered interactions during the interactive conditions to ensure the examiner did not lead the interaction but merely engaged in parallel play with developmentally appropriate descriptions. Important training points for the second evaluator in the room who video recorded the assessment included minimizing the responses if the participant attempted to engage, remaining situationally aware (especially during passive phase) to ensure examiner and examinee safety, and practicing the best positioning for capturing the participant’s actions so that the level of interaction could be accurately viewed on video while minimizing disruptions of interactions with the primary evaluator.

### 2.3. ASIEP-3 Scoring

Following the initial training sessions, the first twenty videos of the ASIEP-3 administrations were scored by three to four raters: at least one of two PhD level psychologists (MB and EC), one master’s level psychometrist (LC), and at least one of two bachelor’s level research assistants (NW and TT). All ratings were compared against the psychologist (MB) who published the original research on the ASIEP-3 in FXS [50]. During the initial phase of scoring, conference calls were used to review the videos, scoring, and questions. All questions were logged and categorized to develop standardized and consistent scoring guidelines. Following co-scoring of the initial videos, the details for scoring the different conditions were systemized for common situations to provide more detailed scoring criteria to optimize interrater reliability. The list of behavioral descriptors that was developed under each condition was reviewed and endorsed by one of the ASIEP’s authors (J.R. Arick, personal communication, 20 April 2019). See Table S1 for details of coding behaviors.

### 2.4. Analysis

The SPSS statistical package version 26 (IBM) was used for statistical analyses. First, a feasibility assessment was conducted to determine the percentage of individuals who participated in the ASIEP-3 and yielded useable data. Useable data met the following criteria: (1) The participant remained in the assessment room for the entire 12 min, (2) administration was standardized (i.e., no additional people were present in the room), and (3) a complete video recording was available for scoring. These usable data are characterized in Section 3.2.1. For interrater reliability calculations, intraclass correlation (ICC) estimates and their 95% confidence intervals were calculated based on the mean-rating (k = 3), absolute-agreement, and 2-way mixed-effects model, with the senior psychologist (MB) acting as the standard for each rater in each ASIEP-3 subdomain. ASIEP-3 scores were evaluated with respect to gender, age, and IQ. Although both the SB-V standard IQ scores (ABIQ and FSIQ) and z-deviation IQ scores (NVIQz, VIQz, and FSIQz) were used in all analyses including cognitive skills, the patterns and relationships were consistent across all IQ score types (standard and z-deviation). To evaluate content validity, correlation analyses were utilized to examine the relationship between ASIEP-3 scores and other measures of social interaction, including the SCQ, SRS-2, Social Avoidance scale of the ABC-C, ADOS-2, and categorical diagnosis of ASD.

An additional exploratory analysis included a rating of the participant’s responses in the switch from the active modeling phase to the passive phase (passive phase switch) using data recorded on the Administration Checklist. During this transition, the examiner withdrew completely from engagement with the participant. Data were collected on whether the participant noticed this switch (yes/no), as well as the emotional quality of this response. If the participant did notice the switch, we collected the following information: (1) when they noticed (within 30 seconds or more than 30 seconds), (2) if there was an observable change in the participant’s emotional state (yes/no), (3) whether the participant attempted to engage with the examiner (yes/no), (4) whether the participant attempted to engage with the recorder (yes/no), (5) if the participant asked questions about the examiner’s switch to a passive interaction (yes/no) and if the questions persisted (yes/no), and (6) whether the participant kept themselves occupied during the passive phase (yes/no). These variables were used to determine if any relationships exist between the passive phase switch and autism symptoms/diagnosis.

Finally, the extremely low Aggression scores on the ASIEP-3 necessitated an additional analysis to examine the representativeness of this FXS sample. We selected four items from the ABC-C that specifically describe aggressive behavior:

Item 4: “aggressive to other children or adults”
Item 10 “temper tantrums/outbursts”
Item 47 “stamps feet or bangs objects or slams doors”
Item 57 “has temper outbursts or tantrums when he/she does not get own way”

We examined the distribution of scores on these four items, as well as the sum of these items (possible score per item range (0–3); total possible for 4 items is 12) in our sample. We then examined these distributions by gender and ASD diagnosis. Finally, the proportion of the sample rated as having aggressive behavior on these four items of the ABC-C was investigated.

## 3. Results

### 3.1. Participant Characteristics

Participants included 27 males and 14 females 1 to 46 years of age (M = 14.4 ± 10.2 years (1.6–46.5)). One male and one female were excluded from the analyses due to a lack of usable scores on the ASIEP (see Feasibility below), leaving 39 participants in total. The sample was primarily Caucasian and non-Hispanic. The group had a range of cognitive abilities (SB-V ABIQ: M(36) = 58.9 ± 17.3, Mdn = 50, range (47–109); SB-V FSIQz score: M(30) = 56.98 ± 21.74, Mdn = 56.04, range (10.48–98.01)), behaviorial symptoms (ABC-C Total M(32) = 37.0 ± 27.3, range (0–98)), adaptive skills (Vineland-3 ABC M(38) = 55.4 ± 22.1, range (20–98)), autism symptoms (SCQ M(32) = 13.6 ± 6.9, range (3–26)); SRS-2 Total M(24) = 67.7 ± 12.1, range (47–88)), and clinical autism diagnoses (ASD 56% (22/39; 20 males, 2 females) (see Table 1). Females had significantly higher IQ scores (*p* < 0.05 for ABIQ and *p* < 0.01 for FSIQ and all deviation IQ scores) and a lower rate of ASD (*p* < 0.001). There were no significant differences between males and females in age, adaptive skills, behavioral problems, or total T-score on the SRS-2, although there were trends toward improved functioning across all measures in females.

### 3.2. ASIEP-3

#### 3.2.1. ASIEP-3 Feasibility and Reliability

**Feasibility**. The ASIEP-3 was successfully administered and completed by all but two of the forty-one total participants enrolled in the study (with one non-compliance due to naptime and one unstandardized administration with the child requiring the parent in the room for completion), resulting in 39 ASIEP-3 Interaction assessments for analysis. Thus, the feasibility for scorable administration among FXS participants in a clinical research setting is estimated to be 95% or greater.

**Administration Reliability.** The senior psychologist (MB) provided feedback to the evaluators, and reliable, accurate administration was achieved after the three practice and two additional ASIEP-3 administrations. The most common areas of feedback were (1) adjusting the examiner’s language to the developmental age of the child, (2) providing directives during the Direct Cues phase at the appropriate intervals and frequency, and (3) ensuring that the participant was exposed to a minimum of three play activities before the switch to the passive phase.

**Scoring Inter-rater Reliability**. Using the senior psychologist (MB) as the standard, across different domains of the ASIEP-3, the ICCs with the bachelor’s-level RAs ranged from 0.24 to 0.85, with the widest 95% confidence interval at −0.35 to 0.64 and the highest and narrowest 95% confidence interval at 0.61–0.94. ICCs with the master’s level psychometrist (LC) and doctoral-level clinical psychologist (EC) ranged from 0.79 to 0.93, with the widest confidence interval at 0.43 to 0.94 for one subdomain, although all other CIs ranged from 0.73 (or above) to 0.98. All four of the ASIEP-3 score codes (Interaction, CIP, No Response, and Aggressive) were highly correlated among the three raters (MB, LC, and EC) (all *r* values > 0.70, all *p* values < 0.001), indicating robust agreement among raters.

#### 3.2.2. ASIEP-3 Score Profiles

A total of 39 ASIEP-3 administrations were analyzed (26 males, 13 females). Overall, there was a wide range of performance in three of the four ASIEP-3 score categories (Interaction, CIP, and No Response), as well as the overall Autistic Interaction Score (AIS) (see Table 2). There was a low incidence of Aggression scores throughout the dataset, resulting in a minimal distribution for the statistical tests. The highest average percentage of ASIEP-3 scores was in the Interaction category for both males (M = 36.5% ± 14.5%) and females (M = 48.7% ± 14.3%), followed by the Constructive Independent Play category (males M = 33.8% + 21.3 and females M = 42.8% ± 16.2).

**Gender**. When evaluating the differences between males and females in the ASIEP-3 categories and for the overall AIS score, males showed a significantly higher average percentage of No Response scores (*p* < 0.001) and overall AIS scores (*p* < 0.01) compared to females, and there was also a trend toward a significantly higher percentage in the Aggressive score domain among males (*p* = 0.084). Females showed a higher mean ASIEP-3 Interaction score (*p* < 0.05) and a lower ASIEP-3 No Response score (p < 0.001) compared to males. The distribution of AIS between males and females differed by such a magnitude that none of the correlations between AIS and the other variables remained significant when examined by gender.

**Age**. For the entire group, the Interaction score was positively correlated with age (rho(39) = 0.409, *p* < 0.01). While there was a negative relationship between age and AIS for the entire sample (rho(39) = −0.340, *p* < 0.05), this was no longer significant when examined by gender.

As the ASIEP-3 was originally developed for use up to age thirteen, we examined the ASIEP-3 scores by age group to determine if the participants outside of the original standardization range had reasonable score profiles. The group was divided into participants thirteen years old and younger (*N* = 22) and those aged fourteen years and older (*N* = 17). There were no significant differences in the distribution of any ASIEP-3 scores between the younger and older participants. When examining potential relationships between age and the ASIEP-3 scores for the younger and older age groups separately, there were no significant relationships. Finally, there were no significant differences in the distribution of gender or autism diagnosis between the younger and older age groups.

**Cognitive Skills.** There were weak to moderate, significant relationships between IQ and the ASIEP Interaction score (ABIQ rho(36) = 0.394, *p* < 0.05), the ASIEP No Response score (FSIQ rho(31) = −0.405, *p* < 0.05), and the ASIEP AIS (ABIQ rho(36) = −0.401, *p* < 0.05). This indicates that those with a higher IQ tend to have higher Interaction scores and lower No Response score and AIS. All deviation IQ scores (NVIQz, VIQz, and FSIQz) were negatively correlated with the ASIEP-3 No Response score (rho(30) = −0.376 to −0.415, *p* < 0.05) such that those with lower IQ scores tended to have more No Response codes during the ASIEP-3.

#### 3.2.3. ASIEP Validity with ASD Diagnosis and other Measures

**Figure 3:**
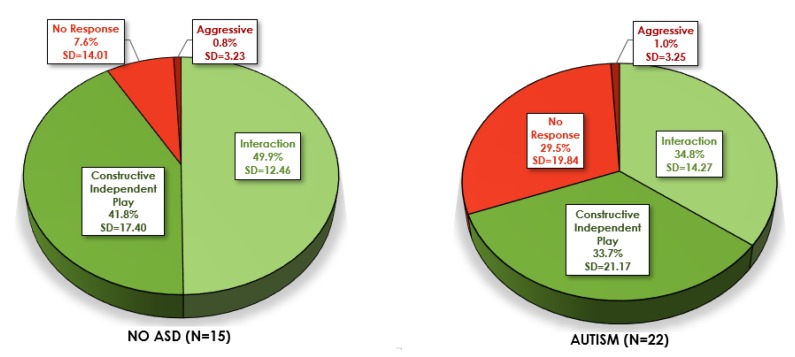
Mean Percentage of ASIEP-3 Interaction Assessment Scores in Fragile X without ASD compared to Fragile X with ASD. ASIEP-3: Autism Screening Instrument for Educational Planning—3rd Edition; ASD: Autism Spectrum Disorder.

**Autism.** ASIEP scores were examined based on a clinical autism diagnosis (see Figure 3). Among those with an autism diagnosis (FXS+ASD *N* = 22), the average percentages of Interaction scores were significantly lower (M(22) = 34.8% versus M(15) = 49.9%, *p* = 0.002), and the No Response scores were significantly higher (M(22) = 29.5% versus M(15) = 7.6%, *p* < 0.001), compared to those with FXS−ASD. Therefore, while the FXS-ASD group earned half of their total score in the Interaction category, those with FXS+ASD earned just over one-third of their scores in the Interaction category. Furthermore, the FXS−ASD group had very low No Response scores (M(15) = 7.6%) compared to the FXS+ASD group who earned one-third of their codes in the No Response category (M(22) = 29.6%). Constructive Independent Play and Aggressive scores were not significantly different based on autism diagnosis (all *p* > 0.10).

**ABC-C**. There were significant positive relationships between ABC-C Social Avoidance and the two ASIEP-3 scores. Both the ASIEP-3 No Response score (rho(32) = 0.384, *p* < 0.05) and ASIEP AIS (rho(32) = 0.368, *p* < 0.05) were correlated with ABC-C Social Avoidance, indicating relationships between the parent-reports of Social Avoidance and the observed behaviors of social avoidance during the ASIEP-3 session.

**SRS-2**. While there were no significant correlations between the SRS-2 Total T-score and any of the ASIEP-3 scores, the SRS-2 Social Motivation T-score was negatively correlated with the ASIEP-3 Interaction score (rho(24) = −0.434, *p* < 0.05). As higher T-scores on the SRS-2 indicate greater autism symptomatology, an increase in Social Motivation deficits was expected to relate to a lower Interaction score.

**SCQ**. The SCQ total score was moderately correlated with ASIEP-3 AIS (rho(28) = 0.435, *p* < 0.05) and approached a significant negative correlation with the ASIEP-3 Interaction score (rho(28) = −0.356, *p* = 0.063).

**Vineland-3**. There were no significant correlations between any of the Vineland-3 scores and any of the ASIEP-3 scores when considering the entire sample, despite the Vineland-3 Socialization standard score being significantly higher among those without autism (FXS−ASD = M(13) = 70.62 + 26.41 versus FXS + ASD = M(24) = 53.58 + 17.10); *t*(35) = 2.382, *p* < 0.05). Among those with an autism diagnosis, the ASIEP-3 Aggression score was negatively correlated with the Vineland-3 Communication standard score (rho(24) = −0.415, *p* < 0.05) and approached a significant negative relationship with the Vineland-3 Socialization standard score (rho(24) = −0.377, *p* = 0.069). The Vineland-3 Socialization standard score was negatively correlated with the ABC-C Social Avoidance score (rho(45) = −0.364, *p* < 0.01) and all of the SRS-2 subscale T-scores (rho(43)= −0.441 to −0.616, all *p* < 0.01).

**ADOS-2.** Contemporaneous ADOS-2 scores were available for a subset (*N* = 10) of participants. All but one of the ADOS-2 administrations resulted in a score in the autism range. In this small homogenous sample, the ADOS-2 Social Affect score did not correlate with the ASIEP-3 scores but did correlate with the parent-report measure of social interaction (SCQ: rho(8) = 0.709, *p* = 0.049). ADOS-2 RRB total was strongly correlated with ASIEP-3 No Response score (rho(10) = 0.642, *p* = 0.045) and ASIEP-3 AIS (rho(10) = 0.635, *p* = 0.049). The negative relationship between the ADOS-2 Total score and the ASIEP-3 CIP score approached significance (rho(10) = −0.584, *p* = 0.073). Most likely due to the small, homogeneous sub-sample and limited score range, the ADOS-2 Comparison score was not significantly correlated with any ASIEP-3 score or other parent-report measures of social interaction.

#### 3.2.4. Exploratory and Ad-hoc Analyses

**Passive Phase Switch.** When considering the ASIEP-3 Administration Checklist results by autism diagnosis (FXS−ASD or FXS+ASD), 87% of those without ASD noticed the switch compared to 58% of those with ASD. Among those that did notice the switch, 90% of those without ASD noticed it immediately, compared to 50% of those with ASD. Those who noticed the switch to the passive phase were significantly older (*t*(37)= −2.530, *p* = 0.016), and their average ASIEP-3 Interaction scores were significantly higher (*t*(37)= −2.506, *p* = 0.017).

**Aggression.** ABC-C (*N* = 32) item 4 (M=.75 ± 0.88; range (0–3)), item 10 (M= 0.94 ± 0.88; range (0–3), item 47 (M = 0.75 ± 0.88; range (0–3)), item 57 (M=1.03 ± 1.03; range (0–3)), and the sum of these four items (M = 3.47 ± 3.09; range (0–12)) showed the range of the FXS phenotype, despite very low ASIEP-3 Aggression scores. These scores were not significantly different based on autism diagnosis (all *p* > 0.05). Further, 15–30% of the participants were rated as moderate or severe in these aggressive behaviors (item 4 = 15.7%, item 10 = 28.1%, item 47 = 21.9%, and item 57 = 34.4%).

## 4. Discussion

### 4.1. Summary of Findings

This pilot study showed that the ASIEP-3 Interaction assessment can be feasibly administered across a wide range of ages and ability levels among males and females with FXS and that this measure produces a range of scores without floor or ceiling effects. The score profiles correlated well with the behaviors that would be expected with a clinical diagnosis of ASD, with the FXS+ASD group showing a lower percentage of time engaged in interactions and more instances of decreased social responses. The score profiles also significantly correlated with other standard measures of social interaction and autism symptoms including the ABC-C Social Avoidance Scale, the SCQ total score, and the SRS-2 Social Motivation T-score. Measures such as the Vineland-3 Socialization domain did not show a significant relationship. This lack of relationship may be related to the broader questions of overall social functioning and social cognition in daily settings on Vineland-3 that capture different aspects than the social interaction features being quantified on the ASIEP-3.

Standardized administration was achieved by all trained research team members who had experience with FXS. Further, the interrater reliability for scoring was very strong among professionals at or above the master’s level, while scoring reliability among the junior research team was more variable. This difference highlights the complexity of assigning behavior codes without training and experience to assess social development among children and adults. In a clinical research setting, this measure could be trained to be administered at multiple sites, with videos scored centrally by trained raters. Centralized scoring has also been utilized in other FXS measures due to the complexity that can accompany the interpretation of FXS behavior [41,71].

We also explored the use of the ASIEP-3 Interaction assessment in an age group beyond the standardization sample with adjustments to the administration materials as described above, and the results support that this measure is valid across the age range in our sample, as there were no significant differences in the distribution of any ASIEP-3 scores between younger and older participants. Because individuals with FXS often demonstrate intellectual disabilities, the chronological ages of those assessed was less important when using this measure. Instead, considering developmental ages afforded the researchers a broader scope in which to assess and systematically analyze responses to a variety of stimuli beyond the cut off of age of thirteen.

Other positive aspects of the ASIEP-3 Interaction assessment include its short administration time, which decreases the burden on the research participants and the research team. The total time per participant ranged from 15–20 min with consideration of set-up time and transition into and out of the room. Further, as the session is designed with toys or objects of interest specific to the participant, the distress of the participant is minimized. Taken together with the validation results above, we determined the ASIEP-Interaction assessment to be a promising and useful measure for the quantification of social interaction skills.

### 4.2. Limitations and Next Steps

This pilot study has limitations and could be improved by a larger sample size. Further, here we only reported results from a baseline cross-sectional sample. The next steps currently underway include an analysis of subsequent administrations at study visits one and two years after this initial administration to determine the longitudinal trajectory of social interactions and test–retest reliability, as well as evaluations over a shorter time period within the context of a typical clinical trial design period of eight to twenty-four weeks. The initial analyses of the Administration Checklist highlight that a lack of recognition of the switch to the passive phase may show high specificity for a diagnosis of ASD, which also deserves further study.

Our sample included participants with few instances of aggressive/negative behaviors during the ASIEP-3 Interaction assessment, which was somewhat surprising given that aggressive behavior can be a significant part of the FXS phenotype [17,72]. Upon ad-hoc analysis, we confirmed that aggressive behavior was represented in our sample by way of the aggressive behavior items on the ABC-C and did not differ based on autism status or gender. Therefore, we do not posit that our sample is biased towards individuals without aggression as an explanation for the low ASIEP-3 Aggression scores. However, it is likely that the unique nature of the ASIEP-3 Interaction tool and this study’s systematic accommodations created a low-stakes environment for the participant, where episodes of aggression are less likely to be observed. Often instances of aggression are demonstrated in situations that cause unexpected stress, anxiety, or frustration [73]. The nature of the Interaction tool is such that all activities are largely play-based, and few demands are directly placed on the participant. The exception to this is the final phase of the assessment, when the participant is given direct cues from the examiner. However, as the final component of the administration, an experienced examiner will have worked to establish a good rapport with the participant, which may make these cues more tolerable. In addition, there is no consequence for a lack of follow through, meaning that the participant is not redirected in any way for not adhering to the cue given. Therefore, the child is free to follow their own lead throughout the entire administration without any redirection. Further, utilization of the Interest Inventory also ensured that appropriate and engaging materials specific to each participant were selected and that activities that may upset the participant or lead to hyperarousal and aggressive behavior could be avoided. As such, the causes for aggression may be largely absent during this specific assessment, which helps to put the lack of aggressive behavior in a broader context.

### 4.3. Clinical Considerations in the Educational Settings and Beyond

In addition to our proposed utilization in clinical trials, this measure was designed to evaluate progress over time in an educational setting and may also be useful in clinical or therapy settings to quantify changes. Despite many research advances in defining the phenotypic characteristics of FXS, this information has yet to reach most educational systems and therapy settings. Many in the FXS field agree that the specific phenotype of FXS provides a strong argument for incorporating etiologic-specific evaluation and intervention strategies and best practice guidelines into educational and therapy programs [74,75,76]. This is supported by the findings that environmental supports for the phenotypic features of FXS contribute significantly to educational outcomes [77]. The potential for ASIEP-3 in an educational setting includes the collection of information to determine the FXS student’s needs and the impacts of the disability when writing an Individual Education Plan (IEP). The assessment results coupled with knowledge of the FXS phenotype could help to establish the types of accommodations necessary for the child to access the general curriculum and/or appropriate activities to make effective progress. Further, this measure was designed with the idea of repeat administrations to assess improvements in social interaction. Indeed, the senior psychologist (MB) in this study has previously used the ASIEP-3 in clinical practice and as a consultant to public school systems to monitor progress. Beyond the Interaction subtest evaluated in this study, the ASIEP-3 comprises four additional subtests that may also prove useful in research and/or play a role in more comprehensive diagnosis, educational, or therapy program placement and planning, as well as the analysis of progress over time.

## 5. Conclusions

Overall, the ASIEP-Interaction assessment shows promise as an efficient, feasible, and valid outcome measure for use in FXS across a broad range of age and ability levels. The potential to capture change over time was established with educational use for idiopathic ASD, and ongoing work from this validation project and future studies will broaden the potential applications of this measure.

## Figures and Tables

**Table 1:** Description of Participants.

	Males	Females	Total	Statistical Results (*t*–Tests and Fisher’s Exact Tests)
**Age *N***	**26**	**13**	**39**	
M (SD)	13.1 (8.1)	17.1 (13.4)	14.4 (10.2)	*t*(37) = −1.177, *p* = 0.247
Range	3.2–29.9	1.6–46.5	1.6–46.5	
Ethnicity: Hispanic *N* (%)	5 (19.2%)	*1* (7.7%)	*6* (15.8%)	*p* = 0.643
Race: Caucasian *N* (%)	*5* (19.2%)	*1* (7.7%)	*31* (81.6%)	*p* = 0.189
**Stanford-Binet-V^ *N***	**25**	**11**	**36**	
ABIQ-M (SD)	54.0 (12.9)	70.1 (21.3)	58.9 (17.3)	*t*(34) = −2.813, *p* = 0.036 *
ABIQ-Range	47–100	47–109	47–109	
FSIQ-M (SD)	46.5 (8.4)	66.7 (17.2)	53.7 (15.5)	*t*(29) = −4.423, *p* = 0.003 **
FSIQ-Range	40–67	40–88	40–88	
FSIQz deviation score- M (SD)	47.2 (17.6)	73.6 (18.2)	57.0 (21.74)	*t*(28) = −3.909, *p* = 0.001 **
FSIQz deviation score- Range	10.5–79.5	35.1–98.0	10.5–98.0	
**Vineland Adaptive Behavior Scales-3**				
Adaptive Composite-M (SD)	51.2 (16.8)	65.6 (30.2)	55.4 (22.1)	*t*(36) = −1.418, *p* = 0.183
Adaptive Composite-Range	20–80	20–98	20–98	
Socialization-M (SD)	55.1 (18.0)	70.2 (27.7)	59.6 (22.1)	*t*(36) = −1.978, *p* = 0.056
Socialization-Range	20–92	20–105	20–105	
Communication-M (SD)	49.2 (23.0)	57.9 (28.4)	52.0 (24.7)	*t*(36) = −1.007, *p* = 0.321
Communication-Range	20–95	20–91	20–95	
**Clinical Autism Diagnosis**				
No ASD- *N* (%)	*4* (16.7%)	*11* (84.6%)	*15* (40.5%)	*p* < 0.001
ASD- *N* (%)	*20* (83.3%)	2 (15.4%)	*22* (56.4%)	
**Aberrant Behavior Checklist**				
Total Score-M (SD)	43.1 (28.1)	25.4 (22.5)	37.0 (27.3)	*t*(30) = 1.806, *p* = 0.081
Total Score-Range	9–98	0–77	0–98	
Social Avoidance Score-M (SD)	2.10 (2.68)	2.18 (3.66)	2.13 (2.99)	*t*(30) = −0.076, *p* = 0.940
Social Avoidance Score-Range	0–8	0–12	0–12	
**Social Communication Questionnaire**				
Total Score-M (SD)	15.4 (6.1)	9.1 (7.2)	13.6 (6.9)	*t*(26) = 2.334, *p* = 0.028 *
Total T-score Range	5–26	3–25	3–26	
**Social Responsiveness Scale-2**				
Total T-score-M (SD)	69.8 (10.7)	62.4 (14.8)	67.7 (12.2)	*t*(22) = 1.3807, *p* = 0.181
Total T-score Range	48–87	47–88	47–88	

* *p* < 0.05 ** *p* < 0.01. ABIQ: Abbreviated Intelligence Quotient; FSIQ: Full Scale Intelligence Quotient; NVIQ: Nonverbal Intelligence Quotient; VIQ: Verbal Intelligence Quotient; ASD: Autism Spectrum Disorder. SB-V ABIQ scores available for 36 participants; SB-V FSIQ and FSIQz scores are available for 19 out of 25 males.

**Table 2:** ASIEP-3 Interaction Assessment Scores.

	Males (*N* = 26)	Females (*N* = 13)	Total (*N* = 39)	Statistical Results (*t*-Tests)
**ASIEP-3 Scores**				
Interaction Percentage-M (SD)	36.5% (14.5)	48.7% (14.3)	40.6% (15.4)	*t*(37) = −2.491, *p* = 0.017 *
Interaction-Range	8.3%–56.3%	16.7%–68.8%	8.3%–68.8%	
CIP Percentage-M (SD)	33.8% (21.3)	42.8% (16.2)	36.8% (20.0)	*t*(37) = −1.336, *p* = 0.190
CIP Range	4.2%–75.0%	6.3%–70.8%	4.2%–75%	
No Response Percentage-M (SD)	27.4% (21.2)	8.5% (12.5)	21.1% (20.7)	*t*(37) = 3.491, *p* = 0.001 ***
No Response-Range	0%–75.0%	0%–35.4%	0%–75%	
Aggressive Percentage-M (SD)	1.4% (3.9)	0% (0)	9.1% (3.2)	*t*(37) = 1.799, *p* = 0.084
Aggressive-Range	0%–12.5%	0%–0%	0%–12.5%	
Autistic Interaction Score-M (SD)	43.6% (14.0)	28.7% (10.2)	38.6% (14.6)	*t*(37) = 3.397, *p* = 0.002 **
Autistic Interaction Score-Range	21%–80%	15%–53%	15%–80%	

* *p* < 0.05 ** *p* < 0.01 *** *p* < 0.001. ASIEP-3: Autism Screening Instrument for Educational Planning; CIP: Constructive Independent Play.

## Supplementary Materials

The following are available online at https://www.mdpi.com/2076-3425/10/4/248/s1, Figure S1: Interest Inventory; Figure S2: ASIEP-3 Administration Checklist; Table S1: ASIEP-3 Detailed Scoring Criteria.
