# Supplementary material for: Evaluating Social Interactions Using the Autism Screening Instrument for Education Planning-3rd Edition (ASIEP-3): Interaction Assessment in Children and Adults with Fragile X Syndrome
Source: Brain Sci. 2020 Apr 22;10(4):248. doi: 10.3390/brainsci10040248 (PMC7226214; doi:10.3390/brainsci10040248)
Supplement: Supplementary file 1 [file brainsci-10-00248-s001.zip › ASIEP Cordeiro Supplementary Table S3 2020_0301.docx]

SUPPLEMENTARY MATERIALS

Table S3. *ASIEP-3 Detailed Scoring Criteria*

| ASIEP Scoring Criteria | |
| --- | --- |
| INTERACTION | - Looking or orienting toward the adult present - Engaging in play or interacting with the adult present - Imitating modeling of the adult present - Initiating conversation about the play or activity - Initiating play with the adult present - Turn taking with the adult present - The participant may be playing with another toy or engaged in an alternate activity while commenting or engaging with the adult |
| CONSTRUCTIVE INDEPENDENT PLAY | - Playing independently with toys, magazines, puzzles stimulus materials - Manipulating the environment to create things - Experimenting with toys such as building towers with blocks, constructing objects with miscellaneous loose parts, playing with playdough, clay, drawing with crayons, putting puzzles together - Putting things into containers or taking them out but not in a repetitive manner - Imaginary play with toys in an appropriate manner (using figures to play in a manner that “tells a story” walking, facing each other and use play figures to talk or do something together, and playing with variety)   *If at any point, the participant repeats a play activity for more than 3 consecutive 10 second scoring intervals, it will then be scored as No Response because it becomes habituated and self-serving (stimulation) and is no longer functional or constructive play.* |
| NO RESPONSE | - Self-stimming with toys/objects, hitting toys together or repeating a non-meaningful action with toys - Engaging in repetitive behavior - i.e. picking up then dumping out, hitting figures together, lining things up, resetting over and over again (3 or more consecutive 10 second scoring intervals) - Self-stimming with own body – i.e. visual inspection, body rocking - Sitting without any observable behavior such as staring off, looking at the stimuli without any interaction or meaningful activity |
