# Supplementary material for: Evaluating Social Interactions Using the Autism Screening Instrument for Education Planning-3rd Edition (ASIEP-3): Interaction Assessment in Children and Adults with Fragile X Syndrome
Source: Brain Sci. 2020 Apr 22;10(4):248. doi: 10.3390/brainsci10040248 (PMC7226214; doi:10.3390/brainsci10040248)
Supplement: Supplementary file 1 [file brainsci-10-00248-s001.zip › ASIEP Supplementary S1 2020_0301.pdf]

In order to have a successful study visit, we would like to learn more about you or your child's interests and dislikes. The information you provide in this form should be about the individual participating in the study.

- If you are an **adult participant** completing this form about yourself, please let us know about your interests and dislikes.
- If you are a **parent/caregiver** whose child is participating, please let us know about your child's interests.

INTEREST INVENTORY

FAVORITE PEOPLE

1. ____________________
2. ____________________
3. ____________________

FAVORITE TV SHOWS OR VIDEOS

1. ____________________
2. ____________________
3. ____________________

FAVORITE DIGITAL APPS

1. ____________________
2. ____________________
3. ____________________

AREAS of INTEREST/COLLECTIONS

1. ____________________
2. ____________________
3. ____________________

FAVORITE FOOD

1. ____________________
2. ____________________
3. ____________________

FAVORITE SPORTS/SPORTS TEAMS

1. ____________________
2. ____________________
3. ____________________

FAVORITE PLAY ACTIVITIES (even those that are perseverative and seem to be nonproductive)

1. ____
2. ____
3. ____
4. ____

Continue to Page 2…

INTEREST INVENTORY CONTINUED

TOPICS TO AVOID (DISLIKES)

1. ____
2. ____
3. ____
4. ____

ACTIVITIES TO AVOID

1. ____
2. ____
3. ____

Are any aids used at school, work or other places (For example: picture schedules)**?**

____

____

____

Anything else we should know to make this visit successful?

____

____

____
