# Supplementary material for: Evaluating Social Interactions Using the Autism Screening Instrument for Education Planning-3rd Edition (ASIEP-3): Interaction Assessment in Children and Adults with Fragile X Syndrome
Source: Brain Sci. 2020 Apr 22;10(4):248. doi: 10.3390/brainsci10040248 (PMC7226214; doi:10.3390/brainsci10040248)
Supplement: Supplementary file 1 [file brainsci-10-00248-s001.zip › ASIEP Supplementary S2 2020_0301.pdf]

ASIEP Checklist

Overall Assessment

Examiner: ________________ **Recorder:** _______________ **Date of Assessment:** ________________

	Activities offered during ASIEP	Activities engaged with during ASIEP
Blocks	☐	☐
Playing Cards	☐	☐
Play House/Tea Party	☐	☐
Garage/Fire station	☐	☐
Big Pegboard	☐	☐
Basic Puzzle	☐	☐
Felt Board/Paper Dolls	☐	☐
Coloring	☐	☐
Crafts – Describe: ______________________________	☐	☐
Board Game – Name: ___________________________	☐	☐
Sensory toys – Describe: ________________________	☐	☐
Book 1 – Name: ________________________________	☐	☐
Other 1 – Describe: _____________________________	☐	☐

1) **Did anything occur that was outside of standard administration/protocol (i.e. another person in room, recording failure, child spoke another language)?** ☐ Yes ☐ No
If yes, describe: ____

2) **Were there reciprocal questions (back and forth conversation) at any point in the assessment?** ☐ Yes ☐ No
If yes, during which phase? *(check all that apply)* ☐ Active Modeling ☐ Passive ☐ Direct Cues

3) **At any point in the assessment did the participant imitate something the examiner or the recorder did or said?** ☐ Yes ☐ No
If yes, did the imitation occur immediately or was it delayed? ☐ Immediate ☐ Delayed
Describe: ____

4) **Were there any aggressive/negative behaviors during assessment?** ☐ Yes ☐ No
If yes, during which phase? *(check all that apply)* ☐ Active Modeling ☐ Passive ☐ Direct Cues
Did you intervene? ☐ Yes ☐ No
(A) What happened directly before the behavior (**Antecedent**): ______________________
(B) Describe the **Behavior**: ____
(C) What happened after the behavior (**Consequence**): ____________________________
If necessary, additional details: ____

5) **Were there any self-stimulatory/self-abuse behaviors during the assessment?** ☐ Yes ☐ No ☐ Maybe
If yes or maybe, during which phase? *(check all that apply)* ☐ Active Modeling ☐ Passive ☐ Direct Cues
Brief description: ____

ASIEP Checklist

Passive Phase

1) **Did subject notice/orient to examiner switching to passive phase?** ☐ Yes ☐ No

   If yes, complete the following:

   - **Is there a change in the subject's emotional state?** ☐ Yes ☐ No

     If yes, check which changes were observed (check all that apply)

     ☐ Emotional ☐Sensitive/Concern ☐Irritability

     ☐ Aggression ☐Tear-up

   - **Does the subject look at the examiner?** ☐ Yes ☐ No

   - **Does the subject look at the recorder?** ☐ Yes ☐ No

   - **Does subject attempt to engage examiner?** ☐ Yes ☐ No

     If yes , how did the subject attempted to engage **examiner**? (check all that apply)

     ☐ Asked Questions ☐Attempted to engage in play

     ☐ Touched examiner ☐Other: ______________________________

     Describe:____

   - **Does subject attempt to engage recorder?** ☐ Yes ☐ No

     If yes , how did the subject attempted to engage **recorder**? (check all that apply)

     ☐ Asked Questions ☐Attempted to engage in play

     ☐ Touched examiner ☐Other: ______________________________

     Describe:____

   - **Does subject ask questions about examiner's switch to passive?** ☐ Yes ☐ No

     If yes, do they persist? ☐ Yes ☐ No

   - **Please describe any other ways the subject noticed/oriented to examiner switching to passive phase** *(If applicable)*:

     ____

2) **Does subject transition independently to a 'play activity' during passive?** ☐ Yes ☐ No

3) **Does subject keep themselves busy during passive phase?** ☐ Yes ☐ Part of the time

   ☐ No ☐ Most of the time

   If most of the time or part of the time , describe:

   ____
